# Effect of global atmospheric aerosol emission change on PM_2.5_-related health impacts

**DOI:** 10.1080/16549716.2019.1664130

**Published:** 2019-09-26

**Authors:** Xerxes Seposo, Kayo Ueda, Sang Seo Park, Kengo Sudo, Toshihiko Takemura, Teruyuki Nakajima

**Affiliations:** a Environmental Health Sciences, Department of Environmental Engineering, Graduate School of Engineering, Kyoto University, Kyoto, Japan; b Environmental Health Sciences, Department of Global Ecology, Graduate School of Global Environmental Studies, Kyoto University, Kyoto, Japan; c School of Earth and Environmental Sciences, Seoul National University, Seoul, South Korea; d Department of Earth and Environmental Studies, Graduate School of Environmental Studies, Nagoya University, Nagoya, Japan; e Climate Change Science Section, Center for Oceanic and Atmospheric Research, Research Institute for Applied Mechanics, Kyushu University, Fukuoka, Japan; f Earth Observation Center, Japan Aerospace Exploration Agency (JAXA), Tsukuba, Japan

**Keywords:** Black carbon, organic carbon, sulfur dioxide, attributable mortality, years life lost

## Abstract

**Background**: Previous research has highlighted the importance of major atmospheric aerosols such as sulfate, through its precursor sulfur dioxide (SO_2_), black carbon (BC), and organic carbon (OC), and their effect on global climate regimes, specifically on their impact on particulate matter measuring ≤ 2.5 μm (PM_2.5_). Policy regulations have attempted to address the change in these major active aerosols and their impact on PM_2.5_, which would presumably have a cascading effect toward the change of health risks.

**Objective**: This study aimed to determine how the change in the global emissions of anthropogenic aerosols affects health, particularly through the change in attributable mortality (AN) and years of life lost (YLL). This study also aimed to explore the importance of using AM/YLL in conveying air pollution health impact message.

**Methods**: The Model for Interdisciplinary Research on Climate was used to estimate the gridded atmospheric PM_2.5_ by changing the emission of SO_2_, BC, and OC. Next, the emissions were utilized to estimate the associated cause-specific risks via an integrated exposure–response function, and its consequent health indicators, AM and YLL, per country.

**Results**: OC change yielded the greatest benefit for all country income groups, particularly among low-middle-income countries. Utilizing either AM or YLL did not alter the order of benefits among upper-middle and high-income countries (UMIC/HIC); however, using either health indicator to express the order of benefit varied among low- and low-middle-income countries (LIC/LMIC).

**Conclusions**: Global and country-specific mitigation efforts focusing on OC-related activities would yield substantial health benefits. Substantial aerosol emission reduction would greatly benefit high-emitting countries (i.e. China and India). Although no difference is found in the order of health outcome benefits in UMIC/HIC, caution is warranted in using either AM or YLL for health impact assessment in LIC/LMIC.

## Background

Exposure to particulate matter (PM) with a diameter ≤ 2.5 μm (PM_2.5_ has been associated with adverse health effects [1,2]. PM_2.5_-related health risks systematically manifest through various biological responses, such as decreased lung function [3], indirect effects on oxidative stress and inflammatory responses [4], and inflammatory cytokine stimulation leading to inflammatory injury [5]. These risks compromise the physiological integrity of the body, particularly affecting susceptible populations, including the elderly [6] and, cardiovascular- [7,8] and respiratory-related risk groups [9].

The magnitude of the problem posed by air pollution, particularly by PM_2.5_, on human health is measured in various health impact metrics, such as attributable mortality (AM), years of life lost (YLL), value of statistical life, value of statistical life year, and disability-adjusted life-years [10]. Cohen, Brauer [11] estimated that 4.2 million deaths were associated with PM_2.5_ exposure, making it the fifth-ranking mortality risk factor based on the Global Burden of Disease study published in Lancet in 2015. Deaths occurring prematurely from an expected life expectancy account for YLL. Apte, Marshall [12] noted that exposure to PM_2.5_ can have life-shortening implications, reducing the mean global life expectancy by 1.4 years. In China, Chen, Ebenstein [13] also observed a similar air pollution-related life expectancy reduction but with a greater magnitude of 3 years. Various health impact metrics has been used to quantify the health impacts of PM_2.5_. Some metrics account for life expectancy, whereas others monetize such benefits. A common aspect of these impact metrics is the emphasis on the magnitude of change in the impact if such interventions were to be implemented. In the case of health impact assessment, for this study, the magnitude of the health impact indicates both potential burden (excess AM/YLL) and potential benefits (averted AM/YLL). The health outcome with the highest-burden also offers an opportunity for the highest benefit if tackled appropriately. Under the premise of constrained resource assumption, maximizing the use of such health resources amidst an array of interventions/activities geared toward decreasing disease burden has become an important aspect of health-care resource prioritization [14].

PM is a complex mixture of anthropogenic and natural materials which are suspended as aerosol particles in the atmosphere [15]. This complex mixture is composed of both primary, those directly emitted to the atmosphere as a result of human activities or natural processes [16], and secondary aerosols, those produced in the atmosphere from precursor gases [17]. Major atmospheric aerosols comprised a combination of both inorganic materials, such as sulfates, nitrates, sea salt and dust, and carbonaceous components such as black carbon (BC) and organic carbon (OC) [18–20]. Atmospheric aerosols affect climate in many ways; some may scatter and lead to cooling, whereas those that absorb light may lead to warming of the planet’s surface [20].

Atmospheric aerosols are emitted from different emission sources, which vary with the technology utilized for growth and development. Major anthropogenic emission sources of BC and OC include biomass and fossil fuel combustion [21,22]. By contrast, SO_2_is emitted from activities related to the shipping industry [23], coal-fired power plants [24,25] and smelters [26]. Recent studies have indicated that atmospheric aerosol plays an important role in climate change through direct and indirect effects. Consequently, increased level of atmospheric aerosol including PM_2.5_, led by rapid economic growth and urbanization would be expected to impact both climate change and human health [27,28]. Changes in these atmospheric aerosols would have varying impacts on PM_2.5_. Similarly, changes in (PM_2.5_) concentration would have accompanying health risks and related health burden. In this study, we intend to address two major questions: 1) ‘If atmospheric aerosols were to change, how large would the PM_2.5_-related health impacts change?,’ and 2) ‘Will the change in the health impact metrics affect the prioritization of health outcome interventions?’.

## Methods

**Figure 1.**
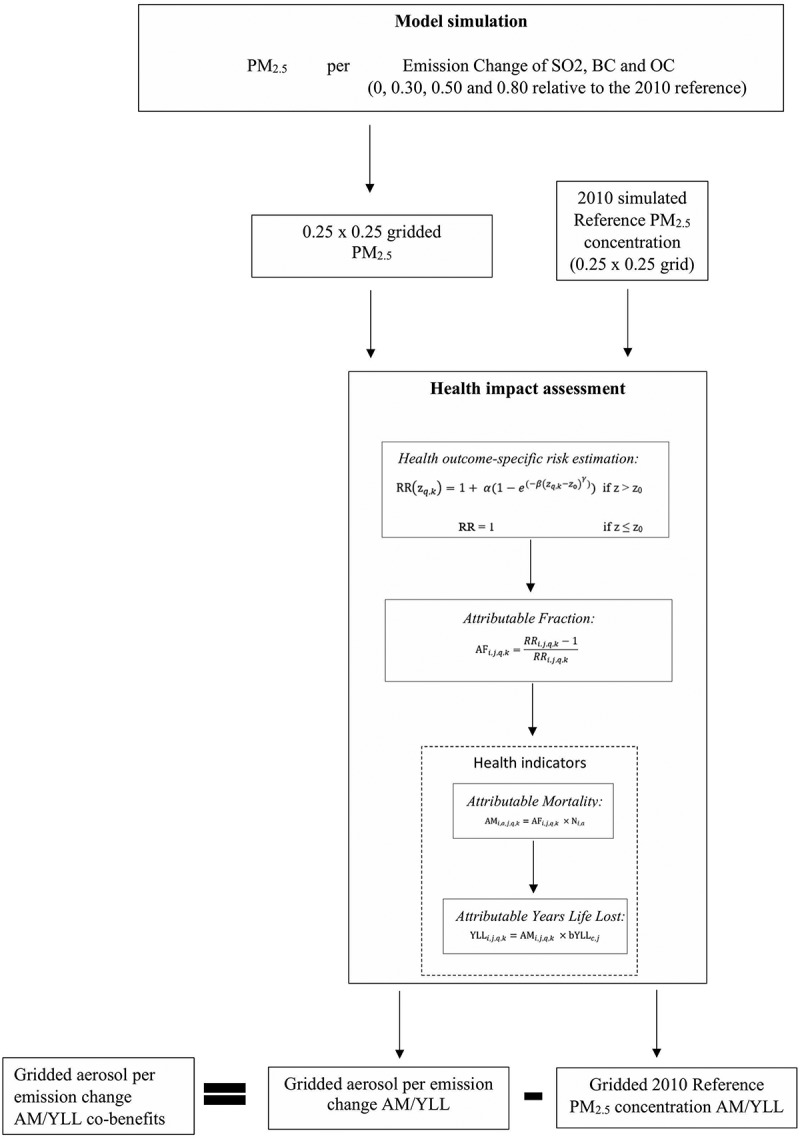
Schematic diagram of the change in the emissions from the model simulation toward health impact assessment.

Grid-based PM_2.5_ concentration is estimated using model simulation at each hypothetical emission level of SO_2_, BC, and OC. The reference year for all atmospheric aerosol emissions was set at 2010, assumed to be a representative of present conditions. Health burden was estimated by using an exposure–response function derived from previous epidemiological studies incorporating information regarding population size and mortality rate of the same period (2010). We assessed the health impact based on the calculated AM and YLL (in Figure 1).

This study has two major components, as highlighted in the schematic diagram, particularly 1) model simulation, and 2) health impact assessment.

### Model simulation

The Model for Interdisciplinary Research on Climate (MIROC), a global atmospheric general circulation model (AGCM) developed by several Japanese institutions [29], was used in simulating global atmospheric aerosol emission. In MIROC, several models were included as a framework of AGCM. For global distribution of the amount of aerosol, the Spectral Radiation-Transport Model for Aerosol Species (SPRINTARS) in MIROC (MIROC-SPRINTARS) calculated the temporal and spatial variation of the amount of aerosol, considering its emission, transport, and sink processes [30]. SPRINTARS considered the aerosol types, by categorizing two carbonaceous aerosols, BC and OC, sulfate with two precursors (SO_2_ and dimethylsulfide), dust from arid surface, and sea salt from the ocean surface. In addition, results from the global chemical model, chemical AGCM for the study of atmospheric environment and radiative forcing (CHASER) [31] were adopted for the formation of aerosol from chemical components.

In this study, the result of MIROC-SPRINTARS was estimated with high spatio-temporal resolution of 0.56° × 0.56°. Global emission of natural-induced aerosols, such as soil dust and sea salt was induced using the original calculation in SPRINTARS based on wind velocity and surface type [32]. The emission of anthropogenically induced aerosols and its chemical precursors were induced from the emission inventories of Hemispheric Transport of Air Pollution version 2 (HTAPv2) at Emission Database for Global Atmospheric Research (EDGAR) [33]. Because the global distribution of these amounts have drastically changed, the reference dataset for anthropogenic emissions was assumed to be the amount in 2010, similar to other studies [34,35]. In addition, HTAPv2 was produced by the monthly-based dataset due to difficulty of emission variation for the interannual scale. Other emission sources, such as biomass burning due to forest fire and field burning, from the database of Global Fire Emission Database Version 3.1, were also included [36]. After model simulation, surface emission of PM_2.5_ was selected using the values in the lowest layer. Furthermore, the gridded data for 0.25° × 0.25° were interpolated after considering the distance between the original and gridded points.

### Health impact assessment: estimating the disease burden due to PM_2.5_

The 0.25° × 0.25° gridded data of the three emission types (SO_2_, BC, and OC) in their respective emission changes (0, 0.30, 0.50, and 0.80 relative to the reference level) were utilized to estimate the grid-specific health indicators; in terms of AM and YLL. The hypothetical emission changes were operationalized to simplify the interpretation of how emission changes would impact the disease burden. The simulated hypothetical emission changes provide varying efforts of reducing aerosol emission from the most stringent, reduction to 0%, to a lesser scale, with reduction to 80% from reference. Climatological studies have similarly conducted varying levels of emission change simulation (i.e. 20% reduction), to provide an insight of how much the reduction will impact countries/regions in terms of aerosol emission/radiative forcing change [37,38].

We used the integrated exposure-response (IER) function developed by Burnett, Pope [39] to estimate the relative risks (RRs), across the grid cells, per health outcome, per emission type, per emission change. Based on previous studies, we used five health outcomes [11,12,39], cerebrovascular diseases (stroke), ischemic heart disease (IHD), chronic obstructive pulmonary disease (COPD), lung cancer (LC), and acute respiratory lung infection (ALRI), in estimating the burden of the disease attributable to exposure (changes in emissions of SO2, BC and OC).

(1) $$\begin{array}{cc} RR\left(z_{q,k}\right)\;=\;1+\alpha \left(1-e^{\left(-\beta {\left(z_{q,k}-z_{0}\right)}^{\gamma }\right)}\right) & if\;z\;>\;z_{0}\\ RR=1 & \;if\;z\leq z_{0} \end{array}$$

where *z_q,k_* is the emission type- and emission change-specific grid-cell mean concentration with varying theoretical minima *z_0_* relative to the health outcomes, and IER emission–response risk curve parameters of *α, β*, and *γ*. To avoid further reiteration and for compactness of notations, we refer to the subscript notations of *q* and *k* as emission type- and emission change-specific attributes, which would mean equivalently the same across equations with the same notations in this study. Four of the five health outcomes, namely stroke, IHD, COPD and LC, are the prevalent air pollution-related mortality observed among adults, whereas ALRI is mostly observed in children 5 years and younger. To account for these variations by age-related health outcomes, we utilized adult mortality rates for stroke, IHD, COPD and LC, whereas under-5 mortality rates were utilized for ALRI. Country-specific 2010 adult population and mortality rates for both men and women aged 20 and older and under-5 population were extracted from WHO [40].

The mean of the predictions of each IER risk curve parameter, per health outcome, previously simulated by Burnett, Pope [39], was used as the IER parameter estimates for this study. After calculating the cause-specific RR per grid cell ($RR_{i,j,q,k}$), these risks were transformed into cause- (*j*) and grid cell (*i*)-specific attributable fraction ($AF_{i,j,q,k}$), which is the proportion of the outcomes attributable to the exposure (Equation 2).

(2) $$AF_{i,j,q,k}=\frac{RR_{i,j,q,k}-1}{RR_{i,j,q,k}}$$

Grid cell-specific baseline mortality (*N_i_*) was derived by multiplying the 2010 grid-specific (*i*), age-group specific (*a*) baseline mortality rates (Table S1) for both adults and children (*bmort_c,a_*) by the 2010 grid cell-specific, age group-specific population (*Pop_i,a_*). Together with the cause- and grid cell-specific attributable fraction (AFs) and the grid-cell and age-group specific baseline mortality, *N_i,a_*, cause- and grid cell-specific attributable mortality (*AM_i,a,j_*) was calculated using Equation 3, as shown below:

(3) $$\begin{array}{rl} & N_{i,a}=bmort_{i,a}\times Pop_{i,a}\\ & AM_{i,a,j,q,k}=AF_{i,j,q,k}\;\times N_{i,a} \end{array}$$

The published data of total cause-specific YLL per country (*YLL_c,j_*) and total cause-specific AN per country (*AN_c,j_*) from WHO [2] were utilized to calculate baseline cause-specific YLL (*bYLL_c,j_*). Cause- and grid-specific YLL (*YLL_i,j,q,k_*) was then estimated by taking the product of the cause- and grid-specific $AM_{i,j,q,k}$ and baseline cause-specific YLL $\left(bYLL_{c,j}\right)$ (Equation 4).

(4) $$bYLL_{c,j}=\frac{YLL_{c,j}}{AN_{c,j}}$$

$$\;\;\;YLL_{i,j,q,k}=AM_{i,j,q,k}\;\times bYLL_{c,j}$$

The health impact by emission change for each grid was assumed as the difference of the emission change-specific estimates of AMs and YLLs for each cause with that of the reference-specific estimates. Subsequently, grid estimates were aggregated to country-specific AMs and YLLs, whereas countries were grouped based on the World Bank Country Classification by Income [41]: low-income country (LIC), low/middle-income country (LMIC), upper-middle-income country (UMIC), and high-income country (HIC). The rationale for the country grouping selection is driven by assumption that countries in the same income group would utilize relatively the same technologies for development and progress. Technology can be captured, to a certain degree, by financial development, which can be subsequently utilized as a determinant for environmental performance (i.e. energy consumption) [42,43]. Similar to other studies [42,44], the groupings’ main intent is to assume that the energy consumption (with the underlying utilization and innovation assumptions) is similar within the same economic (income) groups.

All statistical analyses were performed using R programming [45].

### Order of health benefit based on AN/YLL

Prioritizations in health care are created in response to scarce resources and increased demand of certain health services [46]. Setting up priorities can be based on the ranking of health services or recipients of such services [47]. In this study, we focused on the ranking of recipients (risk population based on the disease burden) resulting from the reduction of air pollution. Norheim, Baltussen [48] emphasized that priority setting can be performed through the ‘realization of (the) potential’, potential in terms of benefits in relation to the intervention/s. If air pollution levels were to be reduced, the change in the disease burden provides decision-makers an overview of the potential benefits across risk populations [48]. We would like to highlight the importance of the health impact, in terms of the disease burden change, and how this health impact can be used as a decision tool for subsequent prioritization of which air pollution-related health outcomes would need greater attention.

**Figure 2.**
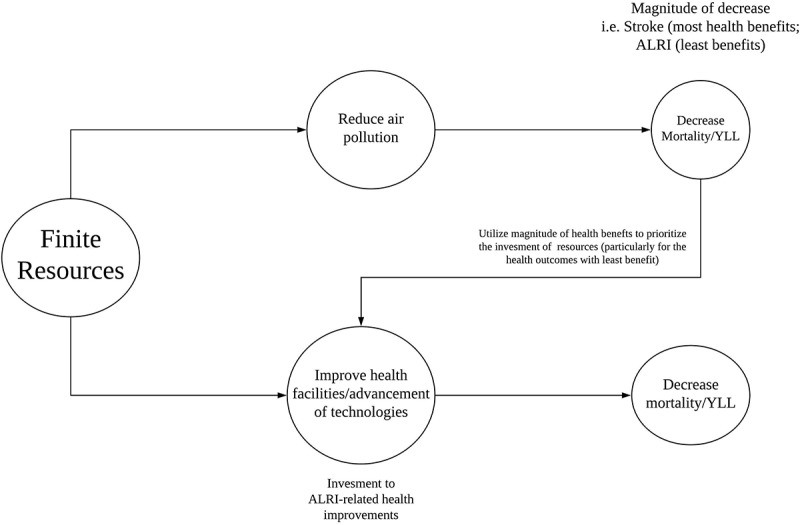
Conceptual health prioritization scheme using the order of the health impact.

To illustrate the importance of the health impact in health outcome prioritization, we assumed a simple scenario whereby the government will have two feasible options to decrease air pollution-related mortality and other related mortalities: a) to allocate toward the initiatives of either reducing air pollution and b) improvement of health facilities/advancement of technologies. The government considers the most efficient health resource used where the gains and losses can be maximized and minimized, respectively. In this study, we only focused on health impact estimation resulting from air pollution reduction. The health outcome with the least health benefit resulting from air pollution reduction can be considered as a priority for health facility improvement, as shown in Figure 2.

In this study, AM and YLL were calculated for each simulated aerosol emission change and the 2010 reference concentration. The difference of the simulated emission change and 2010 reference concentration was considered as health benefit. In this case, the averted AM and YLL serve as potential societal gains/benefits. However, the order of the potential societal gains would largely depend on the health indicator used. Here we provided an additional, albeit simple, schematic scoring, which showcased the importance of utilizing either AM or YLL and their effect on the order of health benefits among the selected health outcomes. The greatest and lowest health benefit will be assigned the value 1 and 5, respectively, arranged in descending order.

The conceptual framework of health prioritization is simplified to highlight the importance of the order of health impact.

## Results

**Figure 3.**
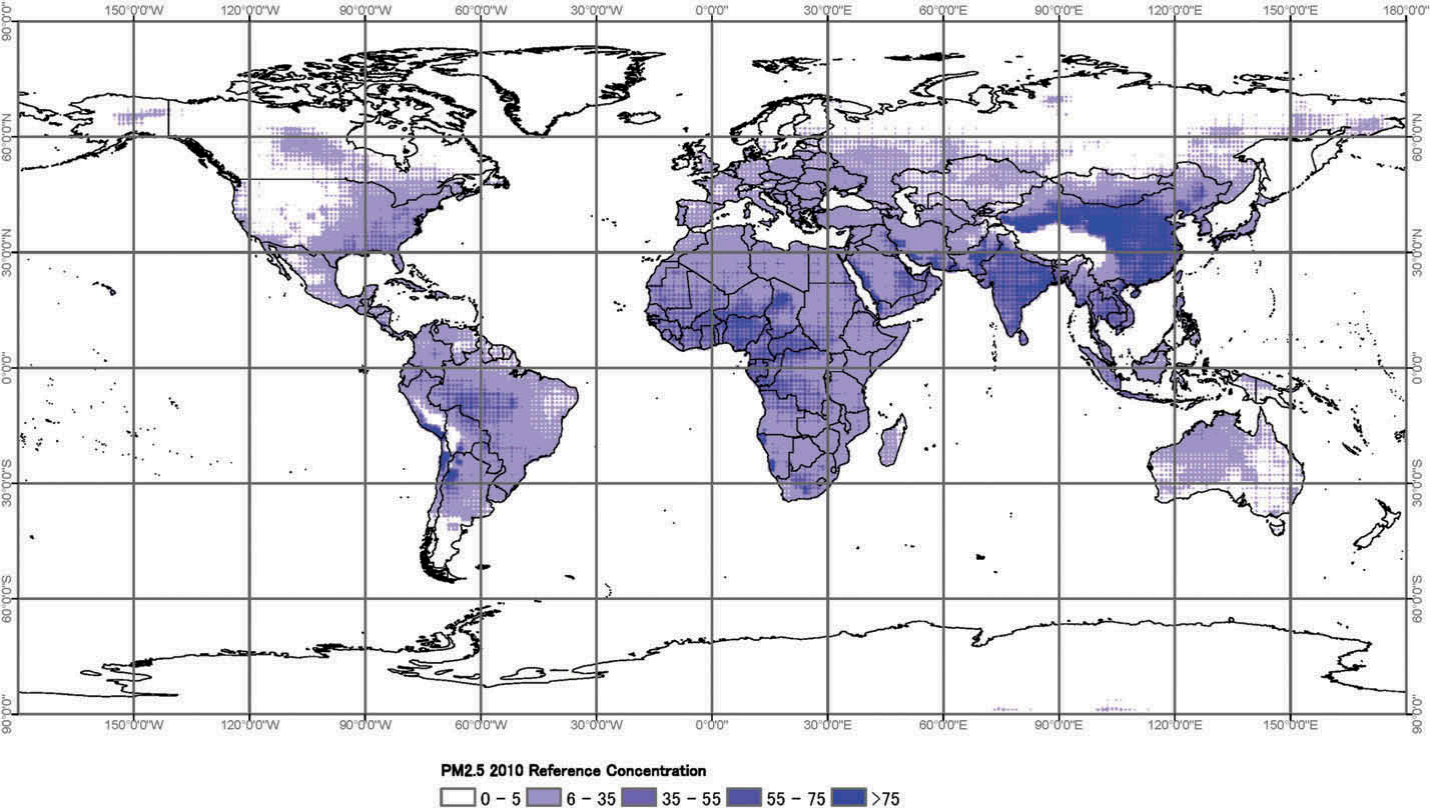
Global reference PM_2.5_ concentration in 2010.

In the interest of compactness of results, only the top 20 countries by order of decreasing interquartile range (IQR) are highlighted in Table 1. China has a wider spread with an IQR of 35.24 µg/m^3^, whereas the IQR of remaining countries was less than twice their respective values compared with that of China’s. Most of the countries were LIC and LMIC countries, except for Saudi Arabia, Kuwait, Brazil, and Malaysia. Similarly, we observe high levels of reference mean concentration of countries among the list, depicted in Figure 3.

**Table 1.** Top 20 countries based on the decreasing interquartile range of gridded reference PM_2.5_ mean (in 2010).

Country	Minimum (µg/m^3^)	Maximum (µg/m^3^)	Mean (µg/m^3^)	Median (µg/m^3^)	IQR (µg/m^3^)
China	0.39	376.84	30.88	23.07	35.24
Pakistan	1.82	112.99	23.97	19.31	16.92
Kuwait	15.39	31.28	22.36	18.87	14.06
Chad	6.87	53.58	19.04	16.55	13.62
Niger	8.00	38.76	18.34	17.4	13.61
Bolivia	0.74	50.4	11.45	12.37	13.14
India	0.61	71.24	24.19	25.39	11.40
Nepal	1.22	43.21	10.84	8.45	11.02
Peru	0.67	211.85	15.25	8.24	10.94
Mongolia	1.10	35.53	9.04	5.35	9.82
Saudi Arabia	7.07	153.27	17.8	12.64	9.70
Myanmar	0.77	26.52	14.74	16.66	9.53
Brazil	1.76	29.59	9.58	8	9.29
Bangladesh	12.11	28.27	20.53	21.39	8.71
Iraq	5.70	72.08	12.4	9.23	8.28
Nigeria	17.72	46.78	27.87	27.47	7.84
Angola	7.06	26.83	14.2	13.19	7.83
The Democratic Republic of Congo	6.00	30.51	17.39	17.86	7.55
Islamic Republic of Iran	4.04	79.49	13.14	7.9	7.55
Malaysia	3.94	26.27	9.04	6.3	7.21

IQR = interquartile range.

The white to the intensified blue gradation indicates the increasing levels of PM_2.5_, with high PM_2.5_ concentration particularly observed in China and India. Apparent darker patches are also observed in both Central African and Western parts of the South American continent.

**Figure 4.**
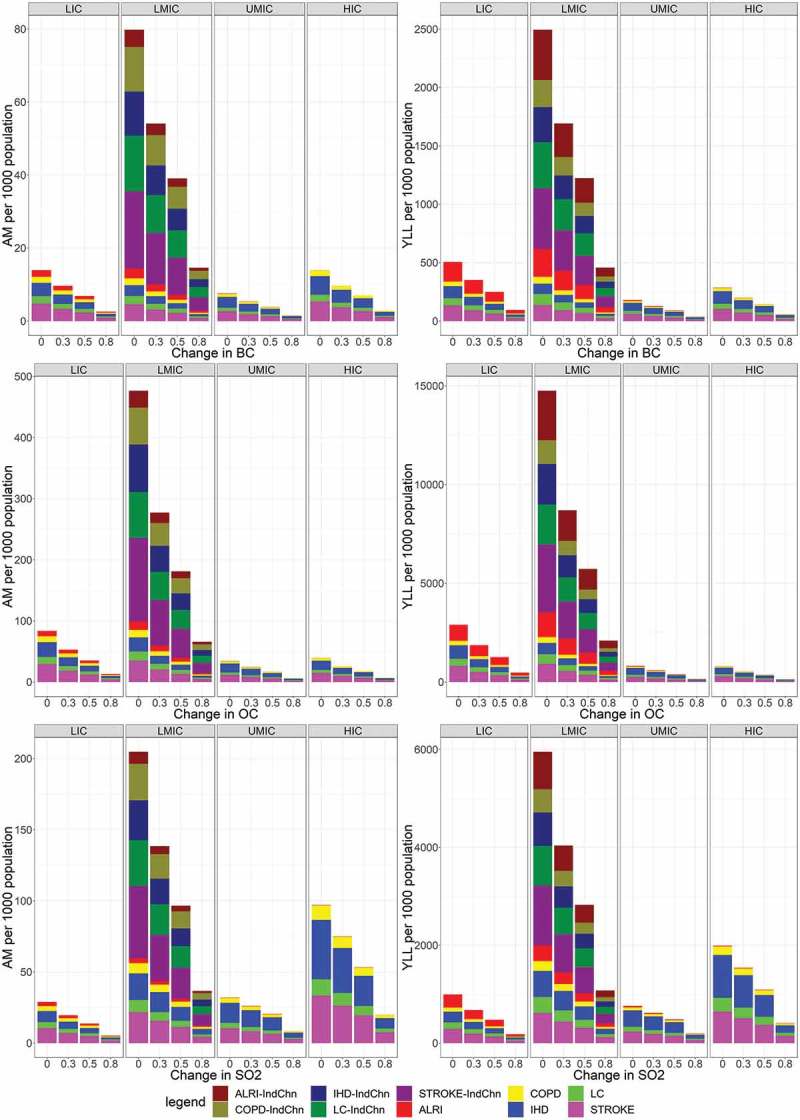
Country income-specific changes in the AM (left panel) and YLL (right panel) per change in BC (upper), OC (middle), and SO_2_ (lower) emission. Dark-colored bars for LMIC indicate the contribution of China and India.

Utilizing the reference concentration in 2010 as baseline, we estimated AM/YLL at baseline and respective simulated changes (0, 0.30, 0.50, and 0.80) for the three major atmospheric aerosols (BC, OC and SO_2_). The estimated AM/YLL at baseline was deducted from the AM/YLL per simulated change per atmospheric aerosol, resulting to estimates of AM/YLL to be interpreted as health benefits if atmospheric aerosols were to be reduced relative to the baseline. In Figure 4, OC reduction yielded the greatest benefit among the two other major aerosol emissions. LMICs would have the highest averted AM and YLL among the country-income groups. The levels of averted AM and YLL in the LMICs, though at least five times greater than other country-income groups, maybe particularly skewed because high-level aerosol-emitting countries such as China and India, were included. Figure 4 shows that the combined benefits of India and China, presented in dark-colored bars, constitute a relatively large proportion among the LMICs, which also contributed to the widened difference in the benefits with that of other country-income groups. By contrast, a consistently higher, yet reduced health benefits apparent among the LMICs, except for AM benefits resulting from reduced SO_2_, would be observed if India and China were excluded. Although HICs have the highest AM benefit in SO2 reduction compared with that for LMIC, with the exclusion of India and China, shifting toward the YLL benefit perspective indicated greater benefits in the LMICs compared with HICs. The change in the magnitude of the benefit in SO_2_ may be partially attributed to change in the health indicator utilized; in this case the shift from AM to YLL. Although the reduction in the atmospheric aerosols resulted to health benefits for either AM/YLL across country income groups, this information may still be insufficient to order the health outcomes based on absolute values of AM/YLL. Instead, the absolute values were rescaled and transformed to a relative scale, in terms of proportion. Hence, the health outcomes can be ordered within health impact metrics, and at the same time, how the order varies between health impact metrics can be compared.

Among the country income groups, LMICs were observed to have the greatest benefits, in terms of averted AM and YLL, particularly in the reduction to 0%. If India and China were to be excluded from the LMICs, the greatest benefits were still apparent in the same country income group, except for the AM benefits resulting from the changes in SO_2_ (lowermost left). Dark-colored bars indicate the separate combined benefits of India and China among the LMICs, whereas lighter colored bars indicate were the respective country-income group, cause-specific benefits.

**Figure 5.**
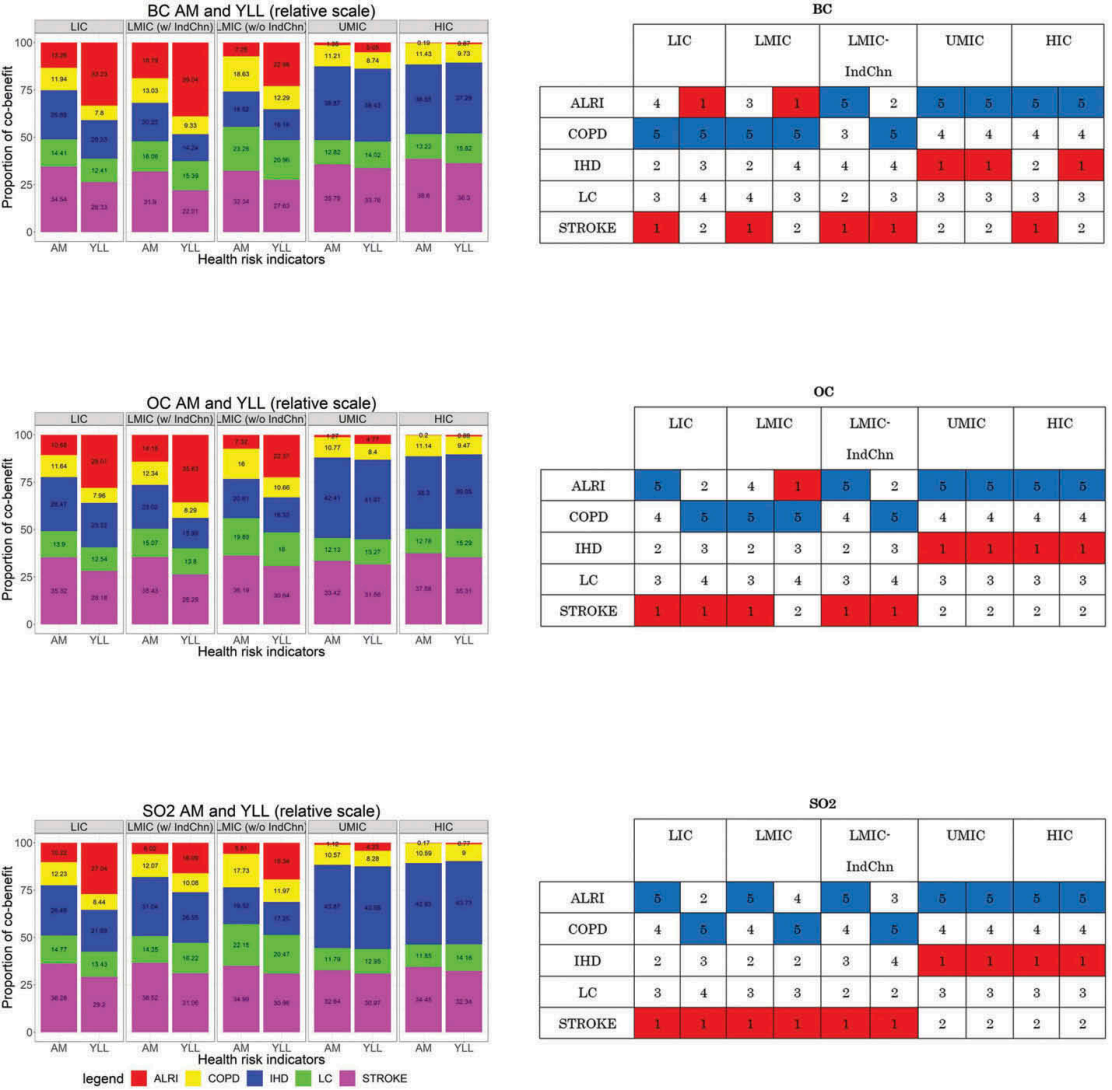
Proportion of reduction to 0% BC, OC, and SO_2_ benefits per health indicator (left) and schematic scoring (right).

Transforming the absolute values of the benefits into relative scales of proportion, the importance of the change of order of health outcomes depending on the proportion of benefits per health indicator was observed (Figure 5, right). After assigning a simple schematic scoring relative to the proportion of the health outcome in the respective health indicators (Figure 5, left), though there is no apparent order across country-income groups, there is a discernable difference between LIC/LMIC (with and without India and China) and UMIC/HIC. Using either AM/YLL did not change the order of the health outcome benefit in UMIC/HIC in different aerosols; however, a large discrepancy between the order in the LIC/LMICs was observed, particularly for BC. Shifting from AM to YLL in BC has changed the order of health outcome benefit in the LIC and LMIC (with India and China); from stroke to ALRI.

Stroke constitute one of the largest proportions of PM_2.5_-related AM and YLL benefits across the country income groups (left). Utilizing either AM or YLL in UMIC and HIC did not alter the order of health benefits; however, the order changed considerably in LIC and LMICs (with and without India and China) as shown in the schematic scoring in the right. Boxes filled with red and numbered with ‘1’ are the health outcomes with the most benefit, whereas those filled with blue and numbered ‘5’ are those with the least benefit.

## Discussion

Changes in the global health indicators, AM and YLL, as well as the associated health benefits, were determined with regard to the simulated emission changes of the two atmospheric aerosols (BC and OC) and a precursor (SO_2_). OC emission change yielded the greatest AM/YLL benefit, particularly for the LMICs. Although both absolute values of AM and YLL benefits may indicate potential gains in different health outcomes, interpretation of the magnitude of these health outcomes were restricted only within the specific health indicator. However, by transforming the absolute AM and YLL benefits into the relative scale, we can observe the relative order of potential gains within the health indicators and determine how this relative order changed between health indicators. The order of health benefits was not consistent across the country income groups and across aerosol; however, an apparent distinction was found between LIC/LMIC when compared with the UMIC/HIC order of health outcomes in either AM or YLL. This study has shown how the changes in atmospheric aerosols may have varying effects on the changes in the health impacts and have subsequently showcased how the utilization of absolute and relative scale magnitude of health indicators affects the order of benefits.

### Atmospheric aerosol emissions

Anthropogenic PM_2.5_ distribution is linked to a variety of aerosol emission sources, which are driven by the technologies being utilized to advance economic development. LMICs such as China and India, are at the forefront of economic growth and industrialization, and in the process of achieving economic development, the decline in its environmental quality is mirrored through the anthropogenic emissions [49], which are apparent in the darker patches shown in Figure 3. The use of biomass fuels in residential areas in India and traditional technologies/practices such as open mass burning for field clearing, has been one of the few sources of the high ambient PM_2.5_ concentrations in the country’s major locations [50]. By contrast, China’s rapid growth and urbanization have led to severe environmental pollution with concentrations beyond healthy air quality levels [51]. Similar to the cultural/traditional practices in India, sub-Saharan Africa owes its increased ambient PM_2.5_ to savanna burning [52], with almost 30% of the tropical biomass being burned in the continent [53].

Each country utilizes various technologies to achieve their current economic status. Alongside the process of maintaining or surpassing their current economic level, these respective technologies have been major contributors to the emission of aerosols, which subsequently affected the current PM_2.5_ concentration. Subject to economic constraints, countries face limited options to reduce one or a few of the aerosol emissions. In this study, the simulated changes in the aerosol emission provided an opportunity for countries to identify which reduction (of aerosol emission) would have the greatest benefits. LMIC countries, such as China and India, being major contributors to global anthropogenic aerosol emission [24], have been observed to have the greatest magnitude of aerosol-related AM and YLL, as shown in Figure 4. China constitutes 14% of the world’s automobiles and its on-road transportation sector contributes a substantial portion of PM_2.5_ [54]. Aside from the transport sector, the coal industry, although regulated through the years, still substantially contributed to aerosol emission [55]. By contrast, India trails behind China as one of the major consumers of coal [56]. Approximately 80% of the carbonaceous aerosols emitted in India were from the use of biomass for energy [57]. This highlights the importance of reducing atmospheric aerosols among high-level aerosol-emitting countries, whereby we expect the greatest benefits. The joint benefits of China and India is even greater than all other countries combined, across different aerosol emissions. Specifically, OC reduction would bring the greatest health benefit, in terms of averted AM and YLL. OC can be reduced by changing from coal to briquettes (BC and OC emission reduction by 80% and 34%, respectively [58]). Otherwise, if the sudden change to briquettes may not be feasible, a gradual introduction of biomass pellets (compressed biomass fuels) may reduce carbon emission from biomass [59].

### Health impact metrics: AM and YLL

Although the reduction of any among the atmospheric aerosols yielded health benefits, these benefits have been observed to vary based on the use of the health impact indicator of interest (AM and YLL), which may also be influenced by country-specific demographics, as shown in Figure 5. As opposed to AM, YLL includes the component of life expectancy, whereby the younger population contributed more YLLs compared with the elderly [60]. Although AM can directly be interpreted as death related to air pollution exposure, YLL is related to life-shortening implications [13]. YLL due to air pollution has been heavily concentrated in the younger population suffering from ALRI, whereas stroke, composed of a majority of the elderly population, account for a relatively smaller portion of YLL. In 2015, lower respiratory tract infections remained as the top leading cause of under-5 mortality with 2.74 million deaths (95% UI: 2.50–2.86 million) [61]. Sub-Saharan African’s Nigeria ranked second in the most number of under-5 mortality at 59,644 (95% UI: 43,761–80,822) [61]. We expected a change in the order of the benefit subject to age-related causes of mortality, particularly the magnification of the YLL benefit among the ALRI population. This magnification was particularly apparent in BC change for LICs, wherein AM benefits for ALRI (red) were relatively smaller than stroke (violet); however, this changed when we used YLL benefit, whereby ALRI benefits were comparatively the same or higher than stroke (Figure 5). This apparent division in the order of health outcomes based on YLL indicated the importance of life expectancy, which was linked to various socio-demographic and inequality variables (i.e. income, labor productivity, old age pension, rural-urban divide) [62,63], in air pollution-related health impact assessment. In China and India, for example, a discrepancy in life expectancy between rural and urban areas was attributed to economic, social life, and sex differences [64,65]. Using either AM/YLL in UMIC/HIC, may not necessarily alter the order of health outcome benefit, which does not alter the health impact message. However, caution should be taken in the utilization of AM/YLL in LICs/LMICs because the order of benefits varied, and this may affect the health impact assessment.

### Limitations of the study

Our study has the following limitations: 1) model simulation, 2) health outcomes, 3) exposure–response function, 4) income grouping, 5) confounding and 6) health indicators. The model simulation is based on robust modeling procedure; however, the simulated change of the atmospheric emission may be limited to a certain extent of the representativeness in doable actual reduction measures. Amidst these restrictions, the simulated changes indicate prospective gains, which can serve as potential target for country-specific strategies. Furthermore, the model simulation may have certain limitations regarding the assumptions utilized in generating the specific aerosol emissions. With regard to the health outcomes, we utilized health outcomes from previous studies [39]. There may be other health outcomes of interest, but we believe that the five major health outcomes would provide sufficient perspective in the PM_2.5_-related health impacts. In this study, we assumed that the exposure–response function of BC, OC and SO2 would follow that of the IER function of PM_2.5_. In the future, these various atmospheric aerosols may have varying exposure–response functions, which we will investigate in future studies. The income grouping utilized in this study may not holistically capture the variations in terms of resources, development and environmental policies, which can affect aerosol emission. More robust and reasonable country grouping/classification is needed in the future to account for the grouping limitations. Moreover, the risk estimates utilized to construct the IER function were a mixture of control/non-control for confounders. Future studies would benefit from air pollution health burden estimation, which accounts for confounding. Finally, the health indicators used were only restricted to AM and YLL. Although other health impact indicators may exist [10], such as value of statistical life, value of statistical life years, and disability-adjusted life years, we have not fully explored the utilization of these health indicators due to limited data availability, but is subject for future research activities.

### Suggestions for future studies

Although the current study has few limitations, future research studies may use these limitations as a guide to further enhance aerosol-related health burden estimation. Specifically, future research may focus on a) policy-linked aerosol mitigation strategies, b) country clustering, and c) utilization of other health indicators. Policy-linked aerosol mitigation strategies would provide insights of what specific interventions can be rolled out in accordance with the global commitments (e.g. Paris agreement). Future studies would also benefit from country clustering, to provide a more coherent overview of the impact of the aerosol mitigation strategies in countries that share the same characteristics. Singling out countries that stand out, without considering countries with lesser changes, may prove to be a limitation in terms of addressing the problem as a global community. Subsequent studies can also explore the monetization of health burden, which can be complemented with the monetization of the implementation of interventions in reducing air pollution-related health burden. The monetization of both the cost of implementation and benefit of implementation may be useful in further tackling the economics of aerosol mitigation strategies.

## Conclusions

This study has shown how reducing specific atmospheric aerosols would have various health benefits. Particularly OC change yielded the greatest benefit for all the country income groups; apparent in the LMICs. Furthermore, aerosol emission reduction would greatly benefit high-emitting countries, such as China and India. Using AM/YLL did not alter the order of health outcome benefits in the UMIC/HIC; however, caution is warranted in using AM/YLL in conveying health impact message, particularly for LIC/LMIC.

## Paper context

Policies have been utilized to target the decrease of atmospheric aerosols, which presumably will affect PM concentration and the consequent associated disease burden. This study demonstrated that OC-reduction would have the greatest health benefit among most, if not all, of the countries. Although the prospective benefits due to the reduced disease burden have been generally positive, caution should be warranted when conducting health impact assessment, particularly for LICs/LMICs because the order of health benefits may vary.
