# Supplementary material for: Effect of global atmospheric aerosol emission change on PM2.5-related health impacts
Source: Glob Health Action. 2019 Sep 26;12(1):1664130. doi: 10.1080/16549716.2019.1664130 (PMC6764381; doi:10.1080/16549716.2019.1664130)
Supplement: Supplemental Material [file ZGHA_A_1664130_SM2354.docx]

Table S1. Cause-specific and All-cause mortality rates (per 1000) per country in 2010

| Country | ALRI | COPD | IHD | STROKE | LC | ALL |
| --- | --- | --- | --- | --- | --- | --- |
| Afghanistan | 0.62994 | 0.121629 | 0.945905 | 0.44496 | 0.027708 | 8.182784 |
| Albania | 0.02374 | 0.172311 | 2.149031 | 1.637617 | 0.344352 | 7.814916 |
| Algeria | 0.080697 | 0.095114 | 1.023912 | 0.384989 | 0.061815 | 4.566293 |
| Angola | 1.02585 | 0.08163 | 0.330733 | 0.292395 | 0.006132 | 10.28355 |
| Antigua and Barbuda | 0.005886 | 0.115431 | 0.943969 | 0.410819 | 0.077257 | 5.86973 |
| Argentina | 0.019757 | 0.550607 | 1.285559 | 0.511293 | 0.239209 | 7.753315 |
| Armenia | 0.033618 | 0.350475 | 2.949577 | 0.940933 | 0.478114 | 9.771215 |
| Australia | 0.001794 | 0.318896 | 1.142813 | 0.508983 | 0.367607 | 6.490858 |
| Austria | 0.000638 | 0.402922 | 2.347243 | 0.627103 | 0.447284 | 9.177934 |
| Azerbaijan | 0.109059 | 0.171303 | 2.327645 | 0.83594 | 0.104237 | 6.546629 |
| Bahamas | 0.036315 | 0.061196 | 0.709761 | 0.42424 | 0.087809 | 5.63753 |
| Bahrain | 0.009096 | 0.053321 | 0.423758 | 0.083864 | 0.048916 | 2.041162 |
| Bangladesh | 0.182472 | 0.335109 | 0.621097 | 0.771213 | 0.054425 | 5.673998 |
| Barbados | 0.004214 | 0.216214 | 1.311118 | 1.031224 | 0.146224 | 10.77363 |
| Belarus | 0.004684 | 0.287866 | 6.981574 | 1.987937 | 0.377296 | 15.0397 |
| Belgium | 0.001342 | 0.61903 | 1.518588 | 0.6837 | 0.643713 | 9.622462 |
| Belize | 0.02447 | 0.129627 | 0.717772 | 0.377803 | 0.089051 | 5.516214 |
| Benin | 0.709957 | 0.134889 | 0.508241 | 0.441425 | 0.004351 | 10.08119 |
| Bhutan | 0.15984 | 0.398359 | 0.903599 | 0.423528 | 0.040365 | 6.034278 |
| Bolivia (Plurinational State of) | 0.193455 | 0.220067 | 0.860373 | 0.455925 | 0.031225 | 6.871868 |
| Bosnia and Herzegovina | 0.00379 | 0.356204 | 2.747054 | 1.969472 | 0.456689 | 10.09063 |
| Botswana | 0.14892 | 0.194169 | 0.565459 | 0.417343 | 0.021934 | 8.64343 |
| Brazil | 0.026811 | 0.289155 | 0.830627 | 0.570805 | 0.127345 | 6.106415 |
| Brunei Darussalam | 0.008888 | 0.145749 | 0.48072 | 0.243194 | 0.116988 | 3.151465 |
| Bulgaria | 0.022441 | 0.545728 | 5.40497 | 3.05977 | 0.454506 | 14.86865 |
| Burkina Faso | 0.514113 | 0.076757 | 0.511202 | 0.288927 | 0.008596 | 10.71085 |
| Burundi | 0.72396 | 0.114243 | 0.391824 | 0.377704 | 0.006827 | 10.47149 |
| Cambodia | 0.204103 | 0.126789 | 0.483434 | 0.667545 | 0.071633 | 6.351677 |
| Cameroon | 0.756797 | 0.144563 | 0.501049 | 0.424001 | 0.008281 | 11.67201 |
| Canada | 0.000943 | 0.374751 | 1.196959 | 0.404486 | 0.569134 | 7.027444 |
| Cape Verde | 0.09229 | 0.172773 | 1.163878 | 0.534065 | 0.015642 | 5.848845 |
| Central African Republic | 0.86687 | 0.167076 | 0.631366 | 0.656092 | 0.008246 | 15.13361 |
| Chad | 1.569643 | 0.121618 | 0.558715 | 0.416457 | 0.004953 | 13.83915 |
| Chile | 0.005394 | 0.275386 | 0.673446 | 0.546532 | 0.164747 | 5.885966 |
| China | 0.025793 | 0.683258 | 1.101082 | 1.299332 | 0.425596 | 6.728079 |
| Colombia | 0.030133 | 0.223394 | 0.924089 | 0.37319 | 0.116685 | 5.642962 |
| Congo | 0.389951 | 0.123054 | 0.517232 | 0.405723 | 0.007161 | 8.598781 |
| Costa Rica | 0.007494 | 0.244237 | 0.765475 | 0.3049 | 0.071989 | 4.799351 |
| Cote d'Ivoire | 0.511892 | 0.153922 | 0.814188 | 0.585013 | 0.008581 | 12.83926 |
| Croatia | 0.001591 | 0.407149 | 3.479535 | 1.777891 | 0.643944 | 12.04208 |
| Cuba | 0.006819 | 0.357184 | 1.821875 | 0.866881 | 0.455566 | 8.043307 |
| Cyprus | 0.000449 | 0.305994 | 1.399697 | 0.528309 | 0.253877 | 6.596308 |
| Czechia | 0.002992 | 0.282547 | 3.239246 | 1.112317 | 0.537631 | 10.13907 |
| Democratic Republic of the Congo | 0.716823 | 0.110413 | 0.368323 | 0.397806 | 0.004688 | 10.92194 |
| Denmark | 0.000766 | 0.695254 | 1.294108 | 0.753122 | 0.709333 | 9.739471 |
| Djibouti | 0.297374 | 0.09289 | 0.54294 | 0.37428 | 0.016424 | 8.308386 |
| Dominican Republic | 0.088527 | 0.098124 | 1.141714 | 0.535463 | 0.097971 | 6.184847 |
| Ecuador | 0.069653 | 0.162905 | 0.434563 | 0.270838 | 0.061853 | 5.052862 |
| Egypt | 0.098906 | 0.179171 | 1.635414 | 0.63555 | 0.050454 | 6.409098 |
| El Salvador | 0.046818 | 0.130027 | 0.991393 | 0.206829 | 0.048169 | 6.412298 |
| Equatorial Guinea | 0.659704 | 0.13069 | 0.581939 | 0.393347 | 0.020367 | 10.85746 |
| Eritrea | 0.38099 | 0.145152 | 0.49537 | 0.514236 | 0.011903 | 8.009008 |
| Estonia | 0.004262 | 0.176622 | 4.495689 | 1.03274 | 0.487132 | 11.84389 |
| Ethiopia | 0.543477 | 0.097644 | 0.451682 | 0.328398 | 0.014175 | 8.418992 |
| Fiji | 0.067355 | 0.147485 | 1.512095 | 0.186983 | 0.036712 | 6.639655 |
| Finland | 0.001111 | 0.228727 | 2.324699 | 0.790376 | 0.413329 | 9.494986 |
| France | 0.000881 | 0.286634 | 1.064076 | 0.554969 | 0.50948 | 8.611149 |
| Gabon | 0.272638 | 0.12511 | 0.573069 | 0.440983 | 0.035317 | 9.371317 |
| Gambia | 0.497457 | 0.109569 | 0.616635 | 0.337422 | 0.009576 | 8.784986 |
| Georgia | 0.018987 | 0.489418 | 4.593829 | 2.624727 | 0.221373 | 12.6399 |
| Germany | 0.00051 | 0.530165 | 2.449462 | 0.779348 | 0.549598 | 10.61877 |
| Ghana | 0.315093 | 0.109056 | 0.662881 | 0.631869 | 0.01172 | 9.076369 |
| Greece | 0.001454 | 0.607956 | 2.275552 | 1.465289 | 0.600978 | 9.524739 |
| Grenada | 0.009419 | 0.176989 | 1.136031 | 0.772587 | 0.084815 | 7.452342 |
| Guatemala | 0.177622 | 0.104092 | 0.415826 | 0.200174 | 0.028531 | 5.22086 |
| Guinea | 0.656025 | 0.154268 | 0.660937 | 0.520006 | 0.007137 | 10.87082 |
| Guinea-Bissau | 0.750861 | 0.116948 | 0.587176 | 0.416207 | 0.009556 | 10.70647 |
| Guyana | 0.049612 | 0.146591 | 1.284088 | 0.864776 | 0.034597 | 7.837105 |
| Haiti | 0.531002 | 0.129998 | 0.916921 | 0.652716 | 0.041104 | 27.18019 |
| Honduras | 0.06975 | 0.137165 | 0.670226 | 0.174856 | 0.032911 | 4.337358 |
| Hungary | 0.002332 | 0.494035 | 4.127871 | 1.410553 | 0.870184 | 13.14373 |
| Iceland | #VALUE! | 0.355171 | 1.365561 | 0.493275 | 0.43913 | 6.267742 |
| India | 0.215144 | 0.590133 | 1.051165 | 0.532387 | 0.05705 | 7.512703 |
| Indonesia | 0.119655 | 0.228137 | 1.191611 | 0.839597 | 0.123127 | 7.073058 |
| Iran (Islamic Republic of) | 0.0484 | 0.135828 | 1.265335 | 0.441401 | 0.054639 | 4.936538 |
| Iraq | 0.209449 | 0.057722 | 0.918834 | 0.328655 | 0.070854 | 4.773329 |
| Ireland | 0.002836 | 0.327508 | 1.144967 | 0.435594 | 0.358571 | 5.859529 |
| Israel | 0.001158 | 0.204727 | 0.716822 | 0.320738 | 0.258127 | 5.314141 |
| Italy | 0.000448 | 0.482559 | 1.783341 | 1.034483 | 0.570435 | 9.793847 |
| Jamaica | 0.009522 | 0.164387 | 0.690642 | 0.818134 | 0.158966 | 6.71246 |
| Japan | 0.00178 | 0.399415 | 1.153366 | 1.021186 | 0.562726 | 9.316294 |
| Jordan | 0.053784 | 0.067685 | 0.753746 | 0.328164 | 0.067646 | 3.887444 |
| Kazakhstan | 0.068016 | 0.360971 | 2.971307 | 1.317056 | 0.267569 | 9.808439 |
| Kenya | 0.350546 | 0.044489 | 0.141054 | 0.143772 | 0.008604 | 7.309535 |
| Kiribati | 0.320257 | 0.150597 | 0.562599 | 0.57452 | 0.08967 | 6.946052 |
| Democratic People's Republic of Korea | 0.074487 | 1.10571 | 1.138532 | 1.686169 | 0.500949 | 8.742517 |
| Republic of Korea | 0.001066 | 0.173997 | 0.472753 | 0.608865 | 0.344609 | 5.154305 |
| Kuwait | 0.009558 | 0.045902 | 0.687852 | 0.257776 | 0.042655 | 2.491776 |
| Kyrgyzstan | 0.175379 | 0.333014 | 2.227106 | 1.036815 | 0.08322 | 7.065877 |
| Lao People's Democratic Republic | 0.453017 | 0.18205 | 0.842185 | 0.691625 | 0.070401 | 7.653163 |
| Latvia | 0.003458 | 0.160826 | 4.897735 | 2.398085 | 0.490699 | 14.21053 |
| Lebanon | 0.00917 | 0.170398 | 2.160307 | 0.316624 | 0.169946 | 6.095391 |
| Lesotho | 0.423221 | 0.359277 | 0.673945 | 0.771553 | 0.0169 | 13.80061 |
| Liberia | 0.541938 | 0.07285 | 0.511549 | 0.339252 | 0.007272 | 9.360264 |
| Libya | 0.030748 | 0.113158 | 1.189038 | 0.335266 | 0.096109 | 4.528934 |
| Lithuania | 0.001752 | 0.264683 | 5.143848 | 1.861181 | 0.424296 | 13.48339 |
| Luxembourg | 0.000437 | 0.433245 | 1.193367 | 0.644358 | 0.424166 | 7.141408 |
| The former Yugoslav Republic of Macedonia | 0.008085 | 0.260299 | 1.578995 | 1.972909 | 0.414414 | 9.246738 |
| Madagascar | 0.457367 | 0.147536 | 0.377871 | 0.530373 | 0.030877 | 7.32464 |
| Malawi | 0.454485 | 0.070523 | 0.297842 | 0.210615 | 0.004285 | 9.8018 |
| Malaysia | 0.008208 | 0.146941 | 1.031755 | 0.426767 | 0.135091 | 4.681551 |
| Maldives | 0.024437 | 0.23216 | 0.856515 | 0.232785 | 0.047583 | 3.330364 |
| Mali | 1.042996 | 0.190734 | 0.590604 | 0.432359 | 0.009847 | 12.39562 |
| Malta | 0.000262 | 0.277455 | 1.995505 | 0.761963 | 0.37665 | 7.618633 |
| Mauritania | 0.641699 | 0.089907 | 0.614115 | 0.3305 | 0.006392 | 8.627663 |
| Mauritius | 0.017114 | 0.304527 | 1.302231 | 0.630525 | 0.108144 | 7.344167 |
| Mexico | 0.04138 | 0.23209 | 0.704138 | 0.274265 | 0.057301 | 5.039869 |
| Micronesia (Federated States of) | 0.142836 | 0.233958 | 0.889423 | 0.637075 | 0.110456 | 6.216246 |
| Republic of Moldova | 0.034131 | 0.404079 | 5.010791 | 1.800572 | 0.261093 | 12.60016 |
| Mongolia | 0.08377 | 0.070349 | 1.292466 | 1.023535 | 0.122126 | 6.407128 |
| Morocco | 0.090374 | 0.129438 | 1.287085 | 0.448613 | 0.102086 | 5.237455 |
| Mozambique | 0.572397 | 0.068078 | 0.300269 | 0.353772 | 0.011814 | 11.56902 |
| Myanmar | 0.266687 | 0.379411 | 0.536935 | 0.909443 | 0.141826 | 8.385187 |
| Namibia | 0.271284 | 0.20185 | 0.594878 | 0.476943 | 0.01954 | 8.570457 |
| Nepal | 0.19608 | 0.447792 | 1.082308 | 0.531351 | 0.082082 | 6.690204 |
| Netherlands | 0.001422 | 0.50507 | 1.168303 | 0.551281 | 0.633879 | 8.156827 |
| New Zealand | 0.005475 | 0.362487 | 1.328071 | 0.566356 | 0.378686 | 6.5538 |
| Nicaragua | 0.103388 | 0.133154 | 0.659222 | 0.244786 | 0.042829 | 4.724819 |
| Niger | 1.11889 | 0.104712 | 0.525958 | 0.36018 | 0.003127 | 11.88387 |
| Nigeria | 1.005778 | 0.110018 | 0.587617 | 0.375554 | 0.005087 | 13.20444 |
| Norway | 0.000502 | 0.49667 | 1.488923 | 0.689699 | 0.467938 | 8.490449 |
| Oman | 0.015697 | 0.034041 | 0.748226 | 0.18645 | 0.019055 | 2.848749 |
| Pakistan | 0.454166 | 0.283594 | 1.258789 | 0.608366 | 0.033387 | 7.866227 |
| Panama | 0.060505 | 0.216637 | 0.724339 | 0.382327 | 0.075333 | 5.002206 |
| Papua New Guinea | 0.316931 | 0.161018 | 0.658332 | 0.488388 | 0.030236 | 7.246779 |
| Paraguay | 0.061387 | 0.134205 | 0.781855 | 0.488173 | 0.098932 | 5.225585 |
| Peru | 0.048655 | 0.15198 | 0.618203 | 0.27703 | 0.080908 | 5.292297 |
| Philippines | 0.165905 | 0.228258 | 1.034891 | 0.73865 | 0.102709 | 6.323843 |
| Poland | 0.002802 | 0.371985 | 3.010854 | 0.991499 | 0.620149 | 9.876256 |
| Portugal | 0.000393 | 0.495214 | 1.275848 | 1.495373 | 0.379442 | 9.980528 |
| Qatar | 0.004559 | 0.015238 | 0.27318 | 0.05456 | 0.036512 | 1.529594 |
| Romania | 0.038692 | 0.351165 | 4.039556 | 2.423834 | 0.476855 | 12.70387 |
| Russian Federation | 0.009379 | 0.231836 | 4.598494 | 2.48952 | 0.428243 | 14.19358 |
| Rwanda | 0.336175 | 0.09974 | 0.258555 | 0.255504 | 0.006562 | 7.168437 |
| Saint Lucia | 0.013615 | 0.278791 | 0.761106 | 0.762301 | 0.098435 | 7.035255 |
| Saint Vincent and the Grenadines | 0.013949 | 0.11391 | 1.354619 | 0.778611 | 0.061057 | 7.359752 |
| Samoa | 0.046714 | 0.173568 | 0.840101 | 0.53416 | 0.049783 | 5.171399 |
| Sao Tome and Principe | 0.277475 | 0.279756 | 0.408041 | 0.45625 | 0.072475 | 6.634367 |
| Saudi Arabia | 0.023927 | 0.058703 | 0.810383 | 0.356001 | 0.025598 | 3.494693 |
| Senegal | 0.4413 | 0.122334 | 0.629449 | 0.364566 | 0.007767 | 7.492365 |
| Seychelles | 0.018362 | 0.205253 | 1.147942 | 0.532361 | 0.146877 | 7.784156 |
| Sierra Leone | 0.838701 | 0.15295 | 0.815455 | 0.598003 | 0.006568 | 14.92084 |
| Singapore | 0.001791 | 0.125455 | 0.916656 | 0.38679 | 0.289153 | 4.548123 |
| Slovakia | 0.005823 | 0.209766 | 3.671973 | 1.091162 | 0.394712 | 9.887695 |
| Slovenia | 0.000917 | 0.259309 | 1.704572 | 0.978401 | 0.561759 | 9.097827 |
| Solomon Islands | 0.159526 | 0.198022 | 0.605275 | 0.511307 | 0.039036 | 4.990062 |
| Somalia | 1.624918 | 0.080831 | 0.459904 | 0.345547 | 0.009824 | 13.17712 |
| South Africa | 0.174063 | 0.280619 | 0.731413 | 0.548721 | 0.121329 | 11.66364 |
| Spain | 0.000867 | 0.504128 | 1.174527 | 0.662396 | 0.456421 | 8.165881 |
| Sri Lanka | 0.013816 | 0.216238 | 1.221868 | 0.574527 | 0.061884 | 6.24729 |
| Sudan | 0.501456 | 0.133821 | 1.245606 | 0.498689 | 0.00953 | 7.879759 |
| Suriname | 0.042707 | 0.115166 | 1.011504 | 0.861796 | 0.114338 | 7.061739 |
| Swaziland | 0.464801 | 0.19647 | 0.462999 | 0.414695 | 0.018698 | 12.6073 |
| Sweden | 0.00107 | 0.415422 | 2.208897 | 0.851451 | 0.399013 | 9.646931 |
| Switzerland | 0.000787 | 0.337331 | 1.565002 | 0.507632 | 0.417287 | 8.017242 |
| Syrian Arab Republic | 0.044191 | 0.062924 | 1.308036 | 0.257267 | 0.09069 | 3.791444 |
| Tajikistan | 0.273577 | 0.168457 | 1.264288 | 0.661254 | 0.03745 | 5.491215 |
| United Republic of Tanzania | 0.524256 | 0.07753 | 0.417144 | 0.240798 | 0.003249 | 9.297374 |
| Thailand | 0.016282 | 0.371197 | 0.787468 | 0.634263 | 0.251493 | 7.264536 |
| Timor-Leste | 0.521181 | 0.122512 | 0.5416 | 0.390413 | 0.135915 | 6.260589 |
| Togo | 0.455452 | 0.137889 | 0.68681 | 0.449234 | 0.006562 | 9.935169 |
| Tonga | 0.051334 | 0.253334 | 0.827141 | 0.445538 | 0.209954 | 5.922389 |
| Trinidad and Tobago | 0.027383 | 0.169867 | 1.505387 | 0.78211 | 0.12642 | 8.587901 |
| Tunisia | 0.028815 | 0.192382 | 1.798797 | 0.657693 | 0.137624 | 6.213007 |
| Turkey | 0.014886 | 0.332667 | 1.161644 | 0.428096 | 0.294422 | 5.750717 |
| Turkmenistan | 0.280779 | 0.069807 | 1.998309 | 0.770659 | 0.088941 | 6.945495 |
| Uganda | 0.524645 | 0.080645 | 0.263364 | 0.250218 | 0.009213 | 9.549653 |
| United Arab Emirates | 0.004382 | 0.046422 | 0.328554 | 0.140287 | 0.015197 | 1.455359 |
| United Kingdom | 0.002478 | 0.53682 | 1.465078 | 0.799442 | 0.556482 | 8.877798 |
| United States of America | 0.002633 | 0.532985 | 1.552872 | 0.42572 | 0.518622 | 8.00826 |
| Uruguay | 0.009821 | 0.566248 | 1.396867 | 0.917922 | 0.407223 | 9.683172 |
| Uzbekistan | 0.117179 | 0.057263 | 2.065443 | 0.629363 | 0.037447 | 5.695512 |
| Vanuatu | 0.119179 | 0.195201 | 0.800931 | 0.505104 | 0.059791 | 4.890104 |
| Venezuela (Bolivarian Republic of) | 0.036988 | 0.157995 | 0.967453 | 0.408993 | 0.126338 | 5.330738 |
| Viet Nam | 0.053126 | 0.224882 | 0.649715 | 0.88444 | 0.204833 | 5.64849 |
| Yemen | 0.365719 | 0.154819 | 1.306393 | 0.561525 | 0.014424 | 6.559457 |
| Zambia | 0.553181 | 0.07708 | 0.342312 | 0.251 | 0.006781 | 9.796591 |
| Zimbabwe | 0.527873 | 0.130169 | 0.407786 | 0.318375 | 0.016897 | 11.96164 |
